# Assessment of Ultra-Early-Stage Liver Fibrosis in Human Non-Alcoholic Fatty Liver Disease by Second-Harmonic Generation Microscopy

**DOI:** 10.3390/ijms23063357

**Published:** 2022-03-20

**Authors:** Takeo Minamikawa, Eiji Hase, Mayuko Ichimura-Shimizu, Yuki Morimoto, Akihiro Suzuki, Takeshi Yasui, Satoko Nakamura, Akemi Tsutsui, Koichi Takaguchi, Koichi Tsuneyama

**Affiliations:** 1 Department of Post-LED Photonics Research, Institute of Post-LED Photonics, Tokushima University, Tokushima 770-8506, Japan; hase@tokushima-u.ac.jp (E.H.); suzuki.akihiro.2@tokushima-u.ac.jp (A.S.); yasui.takeshi@tokushima-u.ac.jp (T.Y.); 2 PRESTO, Japan Science and Technology Agency (JST), Tokushima 770-8506, Japan; 3 Department of Pathology and Laboratory Medicine, Graduate School of Medical Sciences, Tokushima University, Tokushima 770-8503, Japan; ichimura.mayuko@tokushima-u.ac.jp (M.I.-S.); morimoto_yuuki84@yahoo.co.jp (Y.M.); tsuneyama.koichi@tokushima-u.ac.jp (K.T.); 4 Department of Pathology, Kagawa Prefectural Central Hospital, Kagawa 760-8557, Japan; s-nakamura@chp-kagawa.jp; 5 Department of Hepatology, Kagawa Prefectural Central Hospital, Kagawa 760-8557, Japan; a-tsutsui@chp-kagawa.jp (A.T.); k.takaguchi@chp-kagawa.jp (K.T.); 6 Department of Interdisciplinary Researches for Medicine and Photonics, Institute of Post-LED Photonics, Tokushima University, Tokushima 770-8503, Japan

**Keywords:** second-harmonic generation microscopy, non-alcoholic fatty liver disease, non-alcoholic steatohepatitis, fibrosis

## Abstract

Non-alcoholic fatty liver disease (NAFLD) is associated with the chronic progression of fibrosis. In general, the progression of liver fibrosis is determined by a histopathological assessment with a collagen-stained section; however, the ultra-early stage of liver fibrosis is challenging to identify because of the low sensitivity in the collagen-selective staining method. In the present study, we demonstrate the feasibility of second-harmonic generation (SHG) microscopy in the histopathological diagnosis of the liver of NAFLD patients for the quantitative assessment of the ultra-early stage of fibrosis. We investigated four representative NAFLD patients with early stages of fibrosis. SHG microscopy visualised well-matured fibrotic structures and early fibrosis diffusely involving liver tissues, whereas early fibrosis is challenging to be identified by conventional histopathological methods. Furthermore, the SHG emission directionality analysis revealed the maturation of each collagen fibre of each patient. As a result, SHG microscopy is feasible for assessing liver fibrosis on NAFLD patients, including the ultra-early stage of liver fibrosis that is difficult to diagnose by the conventional histopathological method. The assessment method of the ultra-early fibrosis by using SHG microscopy may serve as a crucial means for pathological, clinical, and prognostic diagnosis of NAFLD patients.

## 1. Introduction

Non-alcoholic fatty liver disease (NAFLD) is a complex disease associated with the accumulation of excess fats in the liver, which is considered the most common cause of chronic liver disease [1,2,3,4]. NAFLD has become a more common disease with increasing prevalence in parallel with the global epidemic of obesity-related metabolic syndrome [5,6]. Since NAFLD is closely associated with complex metabolic risk factors, a new definition of metabolic dysfunction-associated liver disease (MAFLD) is recently proposed to describe more appropriately the liver disease related to metabolic dysregulation [7,8]. NAFLD/MAFLD often progresses to non-alcoholic steatohepatitis (NASH), exhibiting severe steatosis, lobular inflammation, hepatocellular ballooning, and liver fibrosis, potentially increasing the risk of cirrhosis and liver cancer. Among the histological features of NAFLD, liver fibrosis is a crucial determinant of NAFLD progression [9,10].

Various diagnostic methods are clinically used to assess fibrosis in the NAFLD liver, including blood tests [11,12], ultrasound/magnetic resonance elastography [13,14,15], and histopathological examination with biopsy [16,17,18]. In the blood test, there are several biomarkers in blood, such as liver enzymes, type IV collagen 7S, hyaluronic acid, and Mac-2 binding protein levels [19,20,21,22]. Ultrasound elastography evaluates the distribution of liver stiffness in relation to the progression of fibrosis by measuring the mechanical properties of the liver as estimated by ultrasound wave propagation properties. Histopathological examination with biopsy is the current gold standard for definitive diagnosis of NAFLD. Tissue sections stained with dyes, such as haematoxylin and eosin (HE) for histological analysis, Masson’s trichrome or Sirius Red staining for fibrosis analysis, evaluate steatosis, and lobular inflammation ballooning, and fibrosis. Although these methods have been clinically applied and have some efficacy in diagnosing NAFLD, it is difficult to characterise the livers of NAFLD at the ultra-early stage in which fibrosis has not yet fully developed. Furthermore, the definitive diagnosis of NAFLD by histopathological examination of biopsy sections requires a highly trained pathologist [23].

In recent years, second-harmonic generation (SHG) microscopy has emerged as a new imaging technique for visualising fibrosis [24,25,26,27,28,29]. SHG emission is observed when an ultrashort laser pulse is irradiated onto a non-centrosymmetric material. Since the major non-centrosymmetric material in biological tissues is collagen, the evaluation of fibrous tissues composed of collagen can be realised by SHG microscopy [24,30,31,32,33,34,35,36,37,38,39,40]. The spatial resolution of SHG microscopy easily achieves 1 μm or less and can be further improved to 200–500 nm by employing appropriate excitation wavelength and scanning structured illumination process [41]. This spatial resolution is sufficient for a histopathological assessment of fibrous tissues. Furthermore, SHG microscopy also has the following unique features. No requirement of staining tissues in SHG measurement reduces the errors in the sample preparation process, leading to quantitative and accurate fibrosis assessment. SHG microscopic assessment can be incorporated into the general histopathological diagnosis process because of the availability of paraffin-embedded tissue sections. Further analysis of the structural maturity of collagen, such as molecular order, packing density, and fibre diameter, is enabled based on SHG intensity and emission directionality [26,42,43,44,45]. Although various researchers evaluate the feasibility of SHG microscopy in liver fibrosis [42,46,47,48,49,50,51], the ultra-early stage of fibrosis of the livers of NAFLD patients is still under investigation.

In the present study, we sought to demonstrate the feasibility of SHG microscopy in the histopathological diagnosis of the livers of NAFLD patients for the quantitative assessment of the ultra-early stage of fibrosis. We examined four representative livers of NAFLD patients, who were clinically diagnosed as ultra-early stages of NAFLD (moderate steatosis, no hepatitis, and no fibrosis), early and advanced stages of NASH. We confirmed the histopathological assessment capability of SHG microscopy in terms of histology of fibrosis and maturity of each collagen fibre. Our result suggests the feasibility of SHG microscopy for assessing liver fibrosis on NAFLD patients, including the ultra-early stage of liver fibrosis that is difficult to diagnose by the conventional histopathological method.

## 2. Results

### 2.1. Clinical and Histopathological Examination of Livers of NAFLD Patients

We examined four representative patients who were clinically diagnosed with NAFLD. Clinical and histopathological features examined by a routine blood test, ultrasound elastography, and histopathological examination are shown in Table 1. Patient 1 and Patient 2 exhibited mild steatosis without hepatitis nor fibrosis as evaluated by histopathological examination. The fibrosis index is based on the four factors (FIB-4 index), the controlled attenuation parameter (CAP) value, the liver stiffness, the FibroScan-aspartate aminotransferase (FAST) score, and the NAFLD activity score (NAS score) exhibited low levels. Thus, Patient 1 and Patient 2 were diagnosed and graded as an ultra-early stage of NAFLD with no apparent evidence of NASH. Patient 3 exhibited mild steatosis and mild hepatitis without apparent fibrosis as evaluated by histopathological examination. The FIB-4 index was in the intermediate range. The CAP value was graded as mild steatosis range (S1). The liver stiffness, the FAST score, and the NAS score exhibited low levels. Thus, Patient 3 was diagnosed and graded as an early stage of NASH. Patient 4 exhibited severe steatosis, mild hepatitis, and moderate fibrosis as evaluated by the histopathological examination. FIB-4 index was in the intermediate range. The CAP value was graded as severe steatosis range (S3). The liver stiffness was higher in the severe fibrosis criterion (F4). The FAST score was higher than the rule-in criterion for significant fibrosis. The NAS score was in the NASH range. Thus, Patient 4 was diagnosed and graded as an advanced stage of NASH.

**Figure 1:**
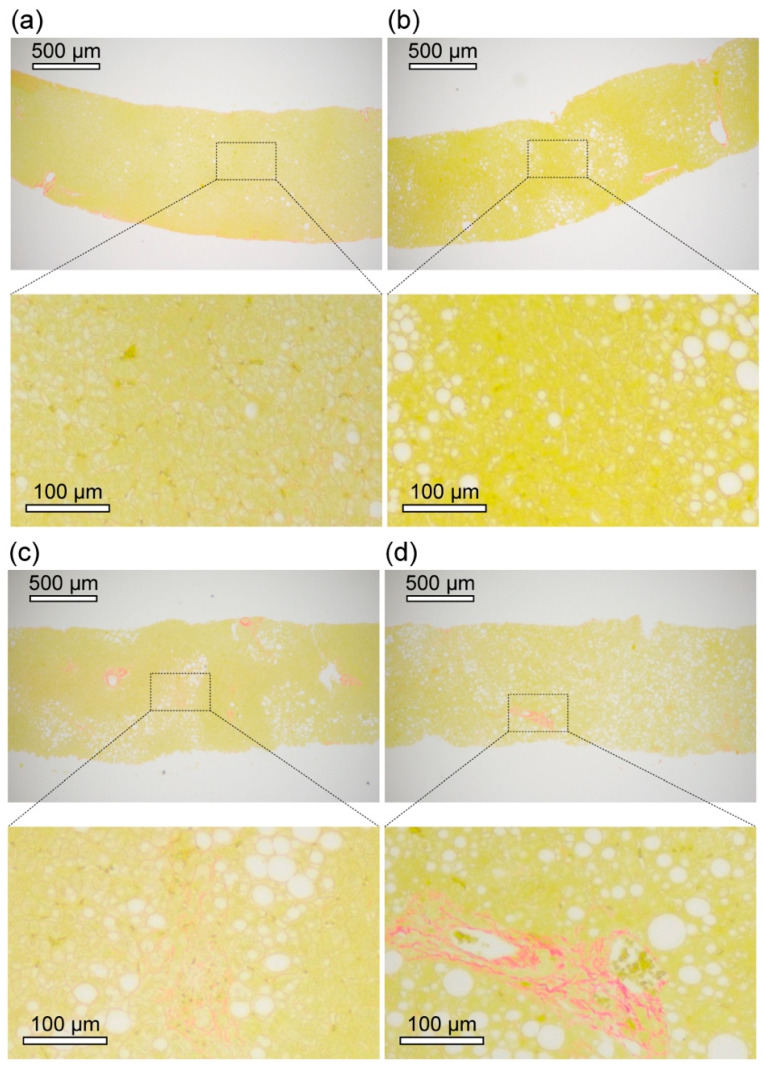
Sirius Red-stained images of the livers of representative NAFLD patients. Sirius Red-stained-images of livers of (**a**) ultra-early stage of NAFLD (Patient 1), (**b**) ultra-early stage of NAFLD (Patient 2), (**c**) early stage of NASH (Patient 3), and (**d**) advanced stage of NASH (Patient 4).

Representative histopathological images of the livers of NAFLD patients are shown in Figure 1. Sirius Red-stained images showed that the microvesicular lipid droplets (LDs; <10 µm in size) were predominantly observed in the liver of Patient 1. In contrast, macrovesicular LDs (>10 µm in size) were abundant and partially localised in the livers of Patient 2 and Patient 3. In Patient 4, the macrovesicular LDs were abundant and spread throughout the liver of Patient 4.

Moderate fibrosis was apparent in the liver of Patient 4 as visualised by the Sirius Red-stained image. In the liver of Patient 3, the slightly stained structures were observed, but there was no apparent fibrosis. Most areas of the liver of the ultra-early stage of NAFLD (Patient 1 and Patient 2) had no apparent fibrosis. In all the livers of patients, the sinusoidal walls seemed to be slightly stained but at a level that would be diagnosed as no fibrosis or suspected artefacts by routine histopathological diagnosis.

### 2.2. Histological Assessment of Liver Fibrosis by SHG Imaging

**Figure 2:**
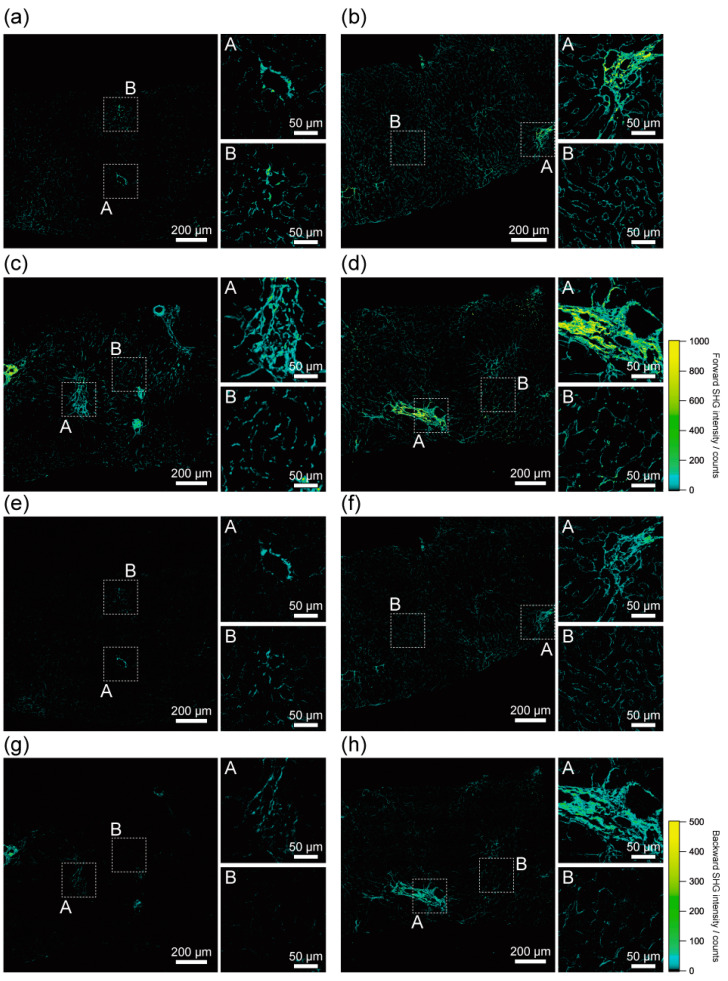
Histological assessment of the livers of NAFLD patients by SHG imaging. Forward-detected SHG images of (**a**) ultra-early stage of NAFLD (Patient 1), (**b**) ultra-early stage of NAFLD (Patient 2), (**c**) early stage of NASH (Patient 3), and (**d**) advanced stage of NASH (Patient 4). Backward-detected SHG images of (**e**) ultra-early stage of NAFLD (Patient 1), (**f**) ultra-early stage of NAFLD (Patient 2), (**g**) early stage of NASH (Patient 3), and (**h**) advanced stage of NASH (Patient 4). Enlarged images of the dashed square regions are also shown.

We investigated the imaging capability of SHG microscopy in the liver of NAFLD patients. Firstly, we examined the histological assessment capability of SHG microscopy, as shown in Figure 2. We obtained SHG images of the livers of NAFLD patients with the forward detection configuration (Figure 2a–d). In all the livers of NAFLD patients, strong SHG signals were obtained at the histologically recognisable fibrous tissues. The fibrous structures, including their thickness, length, and distribution, were clearly visualised by SHG microscopy. Especially in the liver of the advanced stage of NASH (Patient 4), well-organised and networked fibrous tissues were clearly visualised. These results suggest that SHG microscopy has the potential to apply histopathological diagnosis.

Furthermore, SHG images reveal fine structures of fibrous tissues in all livers of the NAFLD patients. Especially in Patient 2, fine fibrous structures appeared to surround the sinusoid throughout the livers. This may indicate the early stage of the development of fibrosis by activated hepatic stellate cells along sinusoids. Interestingly, these fibrous structures were not apparent in the other livers (Figure 2a,c,d). These features of the fine fibrous structures were not apparent in the Sirius Red-stained images (Figure 1a–d). Thus, this sensitive imaging capability of fibrosis is an advantage of SHG microscopy in terms of histological assessment of the livers of NAFLD patients.

Histological assessment capability of SHG microscopy in backward detection configuration was also investigated, as shown in Figure 2e–h. The histological features of SHG images in backward detection configuration almost coincided with that in forward detection configuration. This result indicated that the forward and backward detection configurations are both applicable for the histological assessment of liver fibrosis of NAFLD patients. The results indicated a high degree of freedom in designing the optical system according to applications and the condition of samples in the histological assessment of liver fibrosis. If a thin section sample is available, the forward detection configuration of SHG microscopy is preferred because of the stronger SHG emission in the forward direction due to the coherent generation process of SHG light copropagating with excitation laser light. If forward detection configuration is not available such as with thick liver block or in vivo detection configuration, the backward detection configuration of SHG microscopy is preferred.

### 2.3. Quantitative Assessment of Liver Fibrosis by SHG Intensity

The SHG light intensity strongly depends on the maturity of the fibrous tissue, such as the molecular organisation of collagen molecules. In addition, SHG imaging is highly quantitative because it does not require a staining process that may yield different results depending on the operator. Therefore, SHG light intensity is a suitable candidate as a quantitative index in assessing fibrosis.

**Figure 3:**
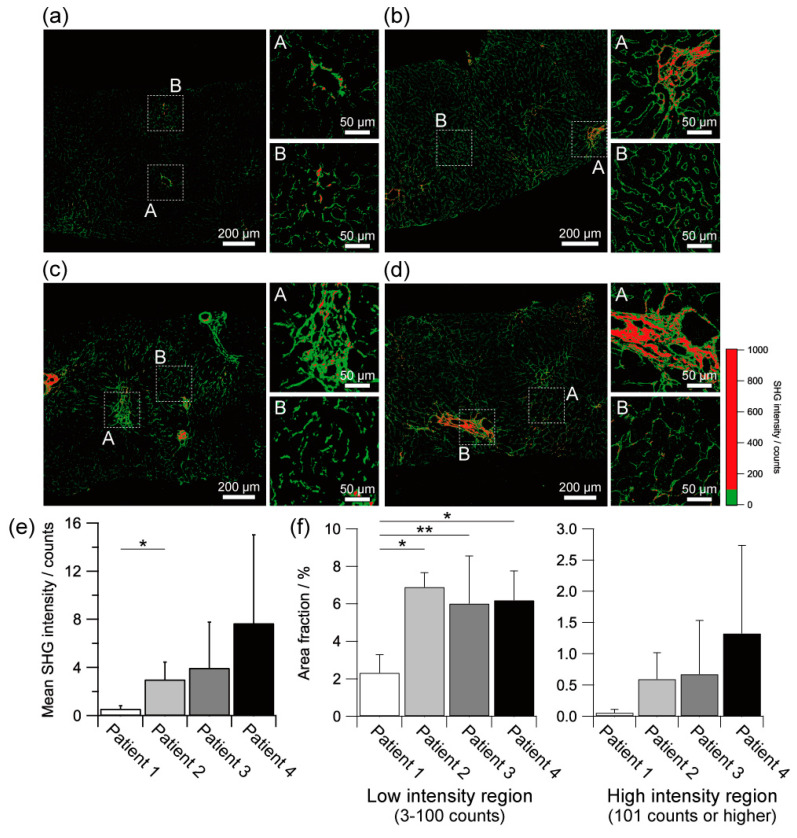
SHG intensity-based analysis of the liver fibrosis of NAFLD patients. Stronger SHG intensity (red) and weaker SHG intensity images (green) of the livers of (**a**) ultra-early stage of NAFLD (Patient 1), (**b**) ultra-early stage of NAFLD (Patient 2), (**c**) early stage of NASH (Patient 3), and (**d**) advanced stage of NASH (Patient 4). (**e**) Mean SHG intensity of each NAFLD patient. (**f**) Area fraction of lower and higher SHG intensity regions of each NAFLD patient. Asterisks indicate a statistically significant difference (* *p* < 0.01, ** *p* < 0.05). Error bar, standard deviation.

As shown in Figure 2a–d, the SHG intensity was strong in the region with histologically well-developed and aggregated fibrous regions. In contrast, the histologically fine fibrous regions exhibited relatively weak SHG intensity. These features are more clearly by obtaining the threshold images as shown in Figure 3a–d. In our experimental condition, we defined the lower SHG intensity region with 3 to 100 counts and the higher SHG intensity region with 100 counts or higher in the forward detection configuration. Interestingly, the strong SHG intensity was also partially observed in a small part of the histologically fine fibrous regions. Since the SHG intensity reflected the molecular organisation within a focal spot (ca. 1 µm^3^ in volume) [56,57,58], these histologically fine fibrous tissues with strong SHG intensity might compose of dense and ordered collagen molecules. These results indicate that the SHG intensity analysis can evaluate the development of fibrosis and the molecular organisation of collagen for liver fibrosis of NAFLD.

The mean SHG intensities tended to be stronger as patients with more advanced NAFLD, but with no significant difference among patients except the comparison between Patient 1 and Patient 2, as shown in Figure 3e. Interestingly, the stronger SHG intensity image visualised not only the histologically well-matured and aggregated fibrous regions but also some of the fine fibrous regions of which the most regions were observed as the weak SHG intensity region. This result suggested that some parts of the fine fibrous tissues were more mature than the others. Furthermore, in the well-matured and aggregated fibrous regions, the stronger SHG intensity image visualised the central structure of the fibrosis, while the SHG images with weaker SHG intensities show the structures at the outer edges. These results suggest that not only the maturity and density of the fibrous tissue inferred from morphological information but also the partial maturity and density of the fibrous tissue can be deduced by SHG intensity.

We also performed an area fraction analysis based on SHG intensity, as shown in Figure 3f. The pixels of the lower SHG intensity region with 3 to 100 counts or the higher SHG intensity region with 100 counts or higher in the forward detection configuration were counted in a region of interest with 500 × 500 µm area and divided by the total number of pixels of the region of interest to determine the area fraction of each SHG intensity region, as a similar manner as described by Hristu et al. [59]. In the lower SHG intensity region, the area fraction of Patient 1 with an ultra-early stage of NAFLD was significantly lower than that of the other patients (*p* < 0.01). The area fractions of Patient 2 with an ultra-early stage of NAFLD, Patient 3 with an early stage of NASH, and Patient 4 with an advanced stage of NASH had no differences. In contrast, in the higher SHG intensity region, there was a tendency for the area fraction to be higher in patients with more advanced NAFLD, although there was no significant difference between patients. These results suggest that the SHG microscopy may have the potential to characterise the ultra-early stage of NAFLD that is hard to precise grading by conventional diagnostic methods.

### 2.4. Molecular Maturation Assessment of Collagenous Fibre by SHG Emission Directionality Analysis

For fibrosis analysis of NAFLD patients in tissue sections, both forward and backward detection configurations can be employed. In such a case, we can perform the SHG emission directionality analysis for a more quantitative maturation assessment of fibrosis. The major SHG light propagates in the forward direction, but some also propagate in the backward direction, as demonstrated in the previous section. Since incoherent backward-propagated SHG light produced by multiple scattering of forward-propagated SHG light is negligible because of thin tissue sections (ca. 2 µm), the relative SHG intensity between forward- and backward-propagated SHG light depends on fibre diameter, packing density, and molecular order of collagen molecules due to the coherence in generating SHG light [58,60].

**Figure 4:**
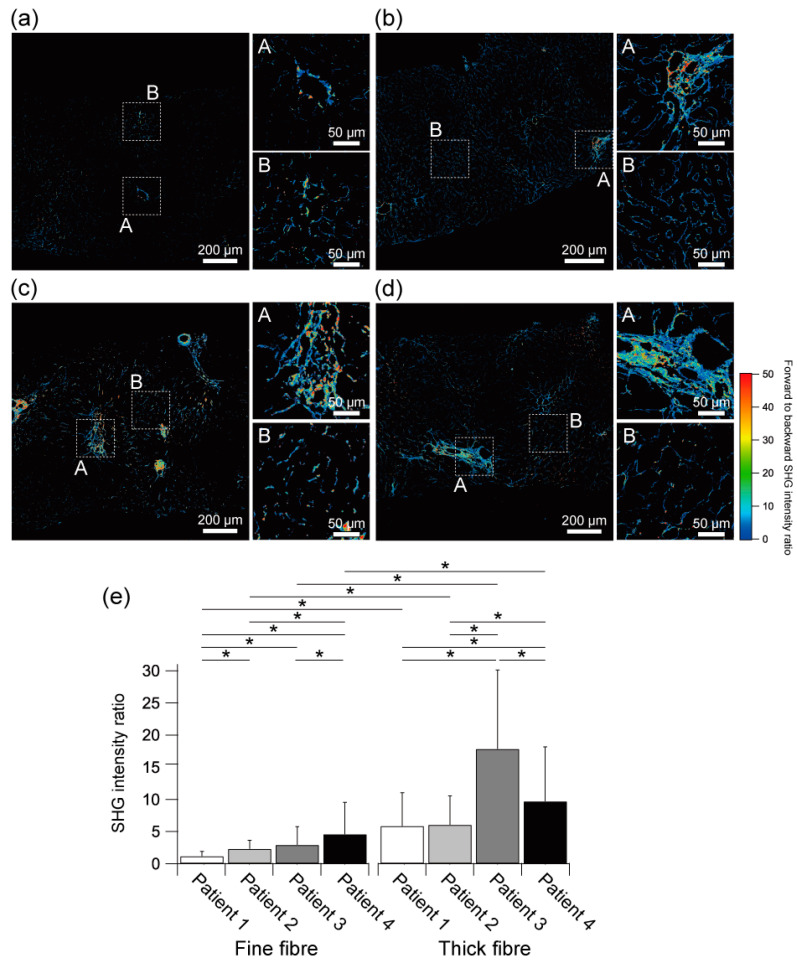
Molecular maturation assessment of collagenous fibre by SHG directionality analysis. SHG intensity ratio images of the livers of (**a**) ultra-early stage of NAFLD (Patient 1), (**b**) ultra-early stage of NAFLD (Patient 2), (**c**) early stage of NASH (Patient 3), and (**d**) advanced stage of NASH (Patient 4). (**e**) Mean SHG intensity ratio of fine (less than 2 µm) and thick (2 µm or thicker) fibrous tissues at each patient. N.A., not applicable. Asterisks indicate a statistically significant difference (* *p* < 0.01). Error bar, standard deviation.

We performed the SHG emission directionality analysis using forward- to backward-detected SHG intensity ratio images, as shown in Figure 4a–d. In the histologically well-developed, well-matured, and aggregated fibrous regions, the forward-detected SHG signal seemed to be stronger than the backward-detected SHG signal, resulting in a high SHG intensity ratio. In contrast, the SHG intensity ratio was relatively lower in the histologically fine fibrous tissues, indicating the relative intensity of backward-detected SHG intensity against forward-detected SHG was higher than that at histologically well-matured and aggregated fibrous regions. This correlation between the SHG intensity ratio and the structural maturity of the fibrous tissue is in suitable agreement with the other reports using rat liver [42] and other tissues [43,44,45]. Furthermore, we found inhomogeneous distributions of SHG intensity ratios in some regions, even for fibrous tissues with a histologically similar appearance. This result indicated that the molecular maturity of fibrous tissues, including fibre diameter, packing density, and molecular order of collagen molecules, was varied depending on collagenous fibres.

Statistical analysis of the mean SHG intensity ratios of fine (less than 2 µm) and thick (2 µm or thicker) fibrous tissues of each patient was shown in Figure 4e. In all the patients, the mean SHG intensity ratios of the thick fibrous tissues were significantly higher than that of the fine fibrous tissues. In the comparison of the fine fibrous tissues among patients, the SHG intensity ratios tended to be higher as patients with more advanced NAFLD with some significant differences. In the thick fibrous tissues, the SHG intensity ratios of the early NASH (Patient 3) and advanced NASH patients (Patient 4) were significantly higher than that of the ultra-early NAFLD patients (Patient 1 and Patient 2). These results suggest that SHG directionality analysis may be one indicator to characterise the progression of fibrosis in NAFLD patients.

However, in the thick fibrous tissues, the mean SHG intensity ratio of the advanced NASH liver (Patient 4) was significantly lower than that of the early NASH liver (Patient 3). As shown in inset A of Figure 4d, the SHG intensity ratio was high at the centre of the fibrous tissue of Patient 4 but was relatively low at the periphery region even with the thick and dense fibres. In contrast, in Patient 3, a high SHG intensity ratio was found even in sparse fibrous tissue structures, as shown in inset A of Figure 4c. As a result, these spatial features of the SHG intensity ratio might have differed in the statistical impact of the mean SHG intensity ratios between Patient 3 and Patient 4. Interestingly, the mean SHG intensity of the thick fibrous tissues of Patient 4 was obviously higher than that of Patient 3, as shown in inset A of Figure 2c,d. SHG intensity and SHG intensity ratio both reflect the molecular maturity of collagenous fibres, such as molecular density, molecular order, and collagenous fibre diameter. The SHG intensity derived by collagenous fibres is mainly determined by the molecular density and molecular order. In addition, if the collagenous fibres are orderly aggregated, constructive interference in the SHG process also enhances the SHG intensity in the forward direction. In contrast, the SHG intensity ratio is affected by the coherent emission process of SHG and the Mie scattering effect, which depends on fibre diameter. Thus, the low SHG intensity but high SHG intensity ratio that appeared in Patient 3 might indicate that the collagen fibres composed of fibrous tissues were sufficiently thick to exhibit a high SHG intensity ratio by Mie scattering, but they are composed of immature collagens with loose and disordered collagen molecules producing low SHG intensity at the molecular level. Conversely, the high SHG intensity but low SHG intensity ratio that appeared in Patient 4 might indicate that the collagen fibres were fine, but they are composed of mature collagens with dense and ordered collagen molecules. These molecular maturation features had the potential to be an indicator to characterise liver fibrosis in NAFLD patients in terms of the SHG directionality analysis.

## 3. Discussion

In the present study, we demonstrated the feasibility of SHG microscopy in assessing liver fibrosis of NAFLD patients at the ultra-early stage. Four representative livers of NAFLD patients, who are clinically diagnosed as an ultra-early stage of NAFLD without hepatitis, early stage of NASH, and advanced stage of NASH were successfully examined in terms of histology and fibrosis maturation by using SHG images, SHG intensity, and SHG emission directionality. Importantly, we revealed that the SHG assessment of liver fibrosis enabled to characterise of the fibrous structures in the livers at not only the advanced stage of NASH but also the ultra-early stage of NAFLD patients that were difficult to diagnose by the general histopathological methods such as using Sirius Red-stained sections.

The progression of liver fibrosis is an essential indicator for assessing NAFLD, whether the liver will progress to cirrhosis and liver cancer. If liver fibrosis is significantly advanced, irreversible fatal damages may occur to the liver, and few effective treatment options are available. In contrast, it has been suggested that the ultra-early stage of liver fibrosis may be reversible. However, liver fibrosis at the ultra-early stage is challenging to detect by general diagnostic methods, such as routine histopathological assessment using stained tissue sections, because the fibrous structure is not sufficiently matured, as in the case of the ultra-early stage (Patient 1 and Patient 2) in this study. This study suggests that SHG microscopy can detect fibrosis with high sensitivity, which is often missed by the conventional diagnostic method. The further development of this study will lead to the diagnosis of liver fibrosis at a reversible stage and provide effective treatment to patients with NAFLD/MAFLD.

For the assessment of liver fibrosis, it is necessary to detect the spatial distribution and the molecular maturity of fibrous tissue with high sensitivity. There are several bioimaging methods focusing on liver fibrosis, such as ultrasound elastography, magnetic resonance (MR) elastography, histopathological imaging with stained tissue sections, and fluorescence microscopy. Ultrasound elastography and MR elastography evaluate the fibrosis in terms of liver stiffness. It is well known that liver stiffness could increase following the progression of fibrosis. The liver stiffness is obtained by evaluating the propagation behaviour of elastic waves through the liver, introduced by ultrasound or mechanical vibration, using ultrasound imaging or MR imaging. These techniques are widely used in clinical practice because they are non-invasive techniques and can be performed several times during the initial diagnosis and follow-up of NAFLD patients. However, the liver stiffness does not significantly change until fibrosis is sufficiently advanced, and thus the detection sensitivity of the liver fibrosis at the ultra-early stage is low. Histopathological imaging using stained tissue sections evaluates fibrosis by staining biopsy-derived liver tissue sections with fibrous tissue-selective dyes. This method is well developed and is a gold standard for the definitive diagnosis of liver fibrosis. However, this approach is based on detecting light transmittance of the dye stained at fibrous tissues, which reduces the detection sensitivity of fine fibrous tissues with little change in light transmittance. Furthermore, the definitive diagnosis of NAFLD using histological features of liver tissues requires a highly trained pathologist. Fluorescence microscopy evaluates fibrosis by detecting the fluorescence emitting from the fluorescent dye selectively stained at the fibrous tissues [61,62]. The dark-field nature of fluorescence microscopy enables the sensitive detection of fibrous tissues stained with fluorescent dyes, even with fine structures. However, the fluorescence dye will become undetectable due to bleaching over time, making it unsuitable for pathological diagnosis where long-term follow-up is required.

By contrast, SHG microscopy can achieve high sensitivity sufficient to assess liver fibrosis, including the ultra-early stage of NAFLD, as demonstrated in this study. Availability of paraffin block tissues sections and no requirement of staining tissues in SHG measurement suggest that the SHG microscopy is suitable for pathological diagnosis where long-term follow-up is required. Furthermore, as shown in this study, the SHG intensity and emission directionality analyses can be easily quantified. If diagnostic indicators of liver fibrosis in NAFLD are obtained with sufficient numbers of clinical trials in further study, SHG microscopy may have the potential to assess liver fibrosis even in an environment where no skilled pathologist is available in the future. In addition, SHG microscopy offers further unique possibilities for assessing liver fibrosis of NAFLD patients as follows. Since the light source and detection method of SHG measurement are less invasive to living organisms, SHG microscopy has in vivo diagnosis capability. Actually, many researchers have investigated in vivo evaluation capability of SHG microscopy or endoscopy with various organs, including the liver [63,64,65,66,67,68]. Although clinically certified SHG systems are limited and no certified system for the liver is currently available, the SHG microscopy has the potential to assess liver fibrosis of NAFLD patients in vivo in the future if the efficacy and safety are confirmed through numerous clinical trials. If clinical applicability is certified, SHG microscopy serves as a useful means for in situ assessment of liver fibrosis during surgery to determine the spatial extent of the liver fibrosis. Three-dimensional SHG imaging can be realised in thick tissue samples or in vivo measurement owing to the optical sectioning capability of the SHG emission derived by nonlinear optical interaction. This feature enables the evaluation of the distribution and orientation of fibrous tissues not only in two dimensions but also in three dimensions, allowing more detailed characterisation of liver fibrosis in NAFLD patients. Dye-stained sections, which are prepared for another histopathological purpose such as HE-stained sections, are also possibly applicable for SHG measurement if necessary [69], while we employed unstained sections to provide the proof-of-principle demonstration in SHG measurement by eliminating as unnecessary effects as possible in this study. If applicable, this feature would be a practical advantage for the future incorporation of SHG diagnosis into routine histopathological diagnosis.

The limitation of this study was the small number of enrolled patients. We examined the typical four patients with NAFLD and did not evaluate the variabilities among patients and biopsy locations. Thus, further studies should examine a large number of patients with various stages of NAFLD to confirm the clinical efficacy of SHG microscopy and determine the criteria for assessing liver fibrosis of NAFLD in the future. Although the limitation exists, this study provides a proof-of-principle demonstration of the feasibility of SHG microscopy for evaluating liver fibrosis on NAFLD patients, including the ultra-early stage of liver fibrosis that is difficult to diagnose by the conventional histopathological methods.

## 4. Materials and Methods

### 4.1. Patients

Liver tissues were collected by needle biopsy from representative four patients who were clinically diagnosed as NAFLD in Kagawa Prefectural Central Hospital from October 2017 to March 2018. One biopsy was performed on each patient. All experiments were performed in accordance with the relevant guidelines and regulations. The study was approved by the Ethics Committee of Kagawa Prefectural Central Hospital (permission no. 1059). For all patients, fully informed consent was obtained after a full explanation of the study design and before the surgery.

### 4.2. Sample Preparation

The obtained liver tissues were immediately fixed with 10% neutral buffered formalin and embedded in paraffin after dehydration with alcohol and clearing with xylene. The paraffin-embedded tissue blocks were sliced into 2 µm-thick sections with a microtome. Three serial sections were obtained. A section was subjected to SHG measurement without any staining. Two sections were subjected to histopathological examination with HE staining or fibrosis assessment with Sirius Red staining.

### 4.3. Clinical and Histopathological Examinations

All the patients were assessed by clinical examination, including routine blood tests, ultrasound elastography, and histopathological diagnosis. A routine blood test was performed for the evaluation of liver enzymes, such as aspartate aminotransferase (AST), alanine aminotransferase (ALT), and platelet count (PLT). By using the age of the patient, AST, ALT, and PLT, we calculated the FIB-4 index, which is one of the indices used for the prediction of liver fibrosis [52,70]. The ultrasound elastography (FibroScan; Echosens, Paris, France) was performed for the evaluation of CAP as the index of steatosis and liver stiffness as the index of fibrosis [71,72]. By using CAP, liver stiffness, and AST, we calculated the FAST score, which is also an index of liver fibrosis [55]. The histopathological examination was performed by using HE- and Sirius Red-stained sections as usual for routine histopathological diagnosis by pathologists in Tokushima University and Kagawa Prefectural Central Hospital. HE-stained sections were subjected to the evaluation of histology of liver tissue, including steatosis and hepatitis. Sirius Red-stained sections were subjected to the evaluation of liver fibrosis. NAS score was calculated by summing the scores for steatosis, lobular inflammation, and ballooning degeneration evaluated by pathologists. The standards for FIB-4 index, CAP value, liver stiffness, FAST score, and NAS score were followed by SVallet-Pichard et al. [52], Karlas et al. [53], Hsu et al. [54], Newsome et al. [55], and Kleiner et al. [73], respectively.

### 4.4. SHG Microscopy

**Figure 5:**
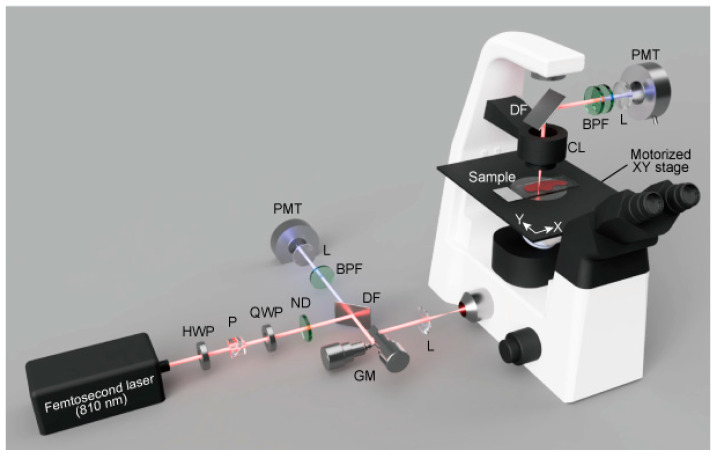
A home-built laser-scanning SHG microscope with a motorised XY stage. BPF, band-pass filter; CL, condenser lens; DF, dichroic filter; GM, galvanometric mirror; HWP, half-waveplate; L, lens; ND, variable neutral density filter; P, polariser; PMT, photon-counting photomultiplier tube; QWP, quarter waveplate.

SHG imaging was performed with a home-built laser-scanning SHG microscope with a motorised XY stage, as shown in Figure 5. A wavelength-tunable femtosecond optical parametric oscillator laser (InSight DeepSee, the tuning range of 680–1300 nm, the pulse duration of ~110 fs, the repetition rate of 80 MHz; Spectra-Physics, CA, USA) operating at 810 nm (SHG wavelength of 405 nm) was employed as the excitation light source. The excitation laser light was focused on a sample through a 60× objective lens (CFI Plan Apo Lambda 60XC, 60×, NA = 0.95; Nikon, Tokyo, Japan) in an inverted optical microscope (Ti2-U, Nikon, Tokyo, Japan). Both forward- and backward-propagated SHG light was detected for imaging. The back-propagated SHG light was collected by the same objective lens, separated from the excitation laser light by a dichroic filter (FF640-FDi02; Semrock, NY, USA) and an optical band-pass filter (FBH405-10; Thorlabs, NJ, USA), and detected by a photon-counting multiplier tube (H8259-01; Hamamatsu Photonics, Shizuoka, Japan). The forward-propagated SHG light was collected by a condenser lens (TI-C-LWD, NA = 0.52; Nikon, Tokyo, Japan), separated from the excitation laser light by a dichroic filter (FF705-Di01; Semrock, NY, USA) and two optical band-pass filters (FBH405-10; Thorlabs, NJ, USA), and detected by a photon-counting multiplier tube (H8259-01; Hamamatsu Photonics, Shizuoka, Japan). Two-dimensional SHG images were obtained by scanning the laser focus with a pair of galvanometric mirrors, in which imaging area, the excitation laser power, and pixel dwell time were set at 137 × 137 µm composed of 256 × 256 pixels, 15 mW on the sample plane and 153 µs, respectively. To acquire a large field-of-view SHG image, we constructed an image mosaic by stitching 100 SHG images obtained by moving a motorised XY stage of the microscope. A single image mosaic was obtained from each biopsied sample that is large enough for the area used in a routine histopathological diagnosis.

### 4.5. Statistical Analysis

Quantitative data were expressed as mean and standard deviation. For the quantitative SHG intensity analysis, randomly selected six areas with 500 × 500 µm in each sample were used. The mean SHG intensity of each area was calculated, then the mean and standard deviation among these areas were obtained. The same six areas were also subjected to the area fraction analysis. The area fraction of SHG intensity in each area was calculated, then the mean and standard deviation among these areas were obtained. For the quantitative SHG intensity ratio analysis, the randomly sampled 50 pixels with the backward-detected SHG intensity of 5 counts or stronger for each fine (less than 2 µm) or thick (2 µm or thicker) fibres were analysed. The fine and thick fibres were extracted using an image mask, in which the median filter with 10 × 10 pixels and thresholding was applied to the forward-detected SHG image. The final extraction results of the fine and thick fibres were also confirmed manually.

Statistical significance was evaluated using Student’s t-test (Figure 3e,f) or Mann–Whitney U test (Figure 4e) depending on the normality of the data distribution. The normality of the data distribution was confirmed by the Shapiro–Wilk test with a significance level of 0.01.

## 5. Conclusions

We revealed the feasibility of the SHG microscopy for assessing liver fibrosis on NALFD patients, including the ultra-early stage of liver fibrosis that is difficult to diagnose by the conventional histopathological methods. Various criteria in the SHG imaging assessment of fibrosis, including histological analysis, SHG intensity, and SHG emission directionality, were effective for a quantitative assessment of liver fibrosis in NAFLD patients. If this method is clinically implemented as a successful diagnosis of liver fibrosis in patients diagnosed as NAFLD/MAFLD in the future, ultra-early diagnosis of the risk of liver fibrosis will be realised before it becomes an advanced NASH for which there has been a lack of effective treatment. Therefore, the assessment method of the ultra-early fibrosis by using SHG microscopy may serve as a crucial means for pathological, clinical, and prognostic diagnosis of NAFLD/MAFLD patients.

## Figures and Tables

**Table 1:** Clinical and histopathological features of NAFLD patients.

	Patient 1	Patient 2	Patient 3	Patient 4
Age	41	42	70	59
Sex	Male	Female	Female	Male
Blood test				
AST/IU L^−1^	43	20	29	63
ALT/IU L^−1^	69	31	31	102
PLT/×10^4^ µL^−1^	21.7	23.7	20.0	22.6
FIB-4 index	1.00 (L)	0.94 (L)	1.88 (I)	1.66 (I)
Ultrasound elastography				
CAP/dB m^−1^	201 (S0)	242 (S0)	253 (S1)	322 (S3)
Liver stiffness/kPa	3.8 (F0)	4.2 (F0)	5.7 (F0)	13.1 (F4)
FAST score	0.186 (L)	0.052 (L)	0.177(L)	0.728 (H)
Histopathology				
Steatosis	+	+	+	++
Hepatitis	-	-	+	+
Fibrosis	F0	F0	F0	F2
NAS score	1	2	2	5
Total diagnosed stage	Ultra-early NAFLD	Ultra-early NAFLD	Early NASH	Advanced NASH

Letters in parentheses indicate the grades evaluated by using each assessment. Grading criteria of FIB-4 index [52]: L, <1.30; I, 1.30–2.67; H, >2.67. Grading criteria of CAP value [53]: S0, <248 dB/m; S1, 248–268 dB/m; S2, <268–280 dB/m; S3, >280 dB/m. Grading criteria of liver stiffness [54]: F0, <6.2 kPa; F1 >6.2 kPa; F2, >7.6 kPa; F3 > 8.7 kPa; F4, >11.8 kPa. Grading criteria of FAST score [55]: L (rule-out criterion for significant fibrosis), <0.35; H (rule-in criterion for significant fibrosis), >0.67. AST, aspartate aminotransferase; ALT, alanine aminotransferase; PLT, platelet count; FIB-4 index, fibrosis index based on the four factors; CAP, controlled attenuation parameter; NAS score, NAFLD activity score.

## Data Availability

The data that support the findings of this study are available from the corresponding author upon reasonable request.
